# Real-life evidence in allergen immunotherapy: Moving forward with mHealth apps 

**DOI:** 10.5414/ALX02343E

**Published:** 2023-03-01

**Authors:** Bernardo Sousa-Pinto, Oliver Pfaar, Jean Bousquet

**Affiliations:** 1 MEDCIDS, Department of Community Medicine, Information and Health Decision Sciences, Faculty of Medicine,; 2 CINTESIS, Center for Health Technology and Services Research,; 3 RISE, Health Research Network, University of Porto, Porto, Portugal,; 4 Section of Rhinology and Allergy, Department of Otorhinolaryngology, Head and Neck Surgery, University Hospital Marburg, Philipps-Universität Marburg, Marburg,; 5 Institute of Allergology, Charité – Universitätsmedizin Berlin, Corporate Member of Freie Universität Berlin and Humboldt-Universität zu Berlin,; 6 Fraunhofer Institute for Translational Medicine and Pharmacology ITMP, Allergology and Immunology, Berlin, Germany, and; 7 University Hospital Montpellier, Montpellier, France

**Keywords:** allergen immunotherapy, allergic rhinitis, mobile health, real-world data

## Abstract

Aim: The efficacy and safety of allergen immunotherapy (AIT) in allergic rhinitis has been classically assessed using randomized controlled trials (RCTs). However, RCTs may have limitations in their external validity, and their evidence may be complemented with that from real-world studies. We aimed to review the mHealth apps that can be used for retrieving real-world data on AIT in allergic rhinitis. Materials and methods: We applied an automatic tool to identify the mHealth apps (available in the Google Play and Apple App stores) that can be used to assess patients under AIT for allergic rhinitis. Apps meeting the inclusion criteria were reviewed, and the corresponding scientific evidence was assessed. Results: We identified five apps with scientific publications in the context of allergic rhinitis: AirRater, AllergyMonitor, MASK-air, Husteblume, and Pollen App. Of those, only MASK-air and AllergyMonitor assessed AIT in patients with allergic rhinitis. MASK-air has enabled the comparison of reported symptoms among patients treated vs. not-treated with AIT. MASK-air has also allowed for the development of combined symptom-medication scores that can be used as endpoints for AIT trials. AllergyMonitor has identified that mobile technology can improve adherence to AIT and is set to support the prescription of AIT for patients with allergic rhinitis by a more precise identification of the pollen season. Conclusion: Mobile health tools allow for the collection of large volumes of real-world data and can be useful for generating hypotheses on AIT. However, such hypotheses require confirmation by epidemiological studies and RCTs.

## Introduction 

Allergen immunotherapy (AIT) is a proven therapeutic option for the treatment of allergic rhinitis and/or asthma [1, 2]. Many international or national practice guidelines have been produced. However, they do not usually propose care pathways based on patient-centered data that can complement randomized controlled trials (RCTs). On the other hand, the sole reliance on evidence from RCTs may be insufficient for the development of patient-centered guidelines, as RCTs tend to narrow the study populations based on specific criteria [3]. The digital transformation of health and healthcare (including mHealth and artificial intelligence) places the patient at the center of the health system and is revolutionizing the practice of medicine [4, 5]. Biomarkers associated with mHealth and clinical decision support systems [6] may change the scope of AIT as they will help monitor the patient’s disease control [7, 8] and enable: (i) patient stratification; (ii) incorporation of evidence from both RCTs and real-world data; (iii) monitoring of the efficacy and safety of targeted therapies (a critical process for identifying appropriate reimbursement), and (iv) implementation of stopping rules [9]. Therefore, in this article, we will discuss real-life evidence in AIT by focusing on mHealth tools that can be used for retrieving and analyzing real-world data associated with AIT use. The potential, findings and limitations of such tools will be discussed. 

## Real-life evidence using mHealth 

To identify which mHealth apps can be used to assess patients under AIT for allergic rhinitis, we searched for rhinitis apps in the Google Play and Apple App stores, via an automatic market research tool recently developed using JavaScript [10]. Over 1,500 apps for allergic rhinitis and rhinosinusitis were identified. However, only 5 apps for rhinitis (AllergyMonitor (TPS software production, Rome, Italy) [11, 12, 13, 14, 15], AirRater (University of Tasmania, Tasmania, Australia) [16], MASK-air (Mobile Airways Sentinel networK for airway diseases, Peercode BV, Geldermalsen, The Netherlands) [4, 7, 17, 18, 19, 20, 21, 22, 23, 24, 25, 26, 27, 28, 29, 30, 31, 32, 33, 34], Pollen App (Medizinische Universität Wien, Vienna, Austria) – patient’s hay fever diary, developed in Austria [35, 36, 37] – and Husteblume (Techniker Krankenkasse, Berlin, Germany) [38], a mobile phone health app developed in Germany as a spin-off of Pollen App including the patient hay fever diary and 2 for rhinosinusitis (Galenus Health [39], Mayo Clinic, Rochester, MN, USA) have published results in the scientific literature. These apps were reviewed for their validation, discovery of novel allergy phenotypes, optimization of the pollen season, novel approaches in diagnosis and management (pharmacotherapy and AIT) and for their adherence to treatment (a more detailed characterization of the different apps can be found in Antó et al. [10] and Sousa-Pinto et al. [40]; Table 1 provides a summary of this characterization). We observed that published evidence demonstrates the potential of mHealth apps to advance in the characterization, diagnosis, and management of rhinitis and rhinosinusitis patients, but also found that only MASK-air and AllergyMonitor have been used in AIT [11, 41]. 

### MASK-air as an example 

MASK-air is a mobile health app that assesses the daily control of allergic rhinitis. It has been freely available since 2015 in the Apple App and Google Play Stores [44] and is currently available in 28 countries. MASK-air has been classified as a Good Practice of DG Santé for digitally enabled, patient-centered care in rhinitis and asthma multimorbidity [45]. It is MDR Class IIa registered and fully complies with the General Data Protection Regulation (GDPR) [46]. 

**Figure 1:**
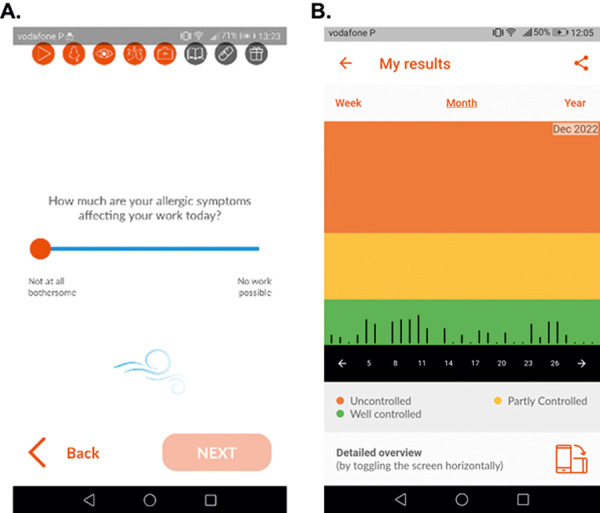
Example of a visual analogue scale in the MASK-air app (A) and a screenshot of the dashboard indicating the patient’s monthly control of allergic rhinitis (B).

In MASK-air, users are requested to report their daily control of allergic rhinitis and asthma by (i) filling in a daily questionnaire comprising 6 visual analogue scales (VASs) (Table 2) (Figure 1) and (ii) entering their daily treatments (medication or AIT). Medication can be entered using a regularly-updated scroll list that contains country-specific and over-the-counter medications (the International Nonproprietary Names classification is used for drug nomenclature) [47]. 

In addition to the symptom and medication daily monitoring questionnaire, MASK-air users may respond (albeit in a non-mandatory way) to several other validated questionnaires, including the EQ-5D-5L [48], the Control of Allergic Rhinitis and Asthma Test (CARAT) [49] and the Work Productivity and Activity Impairment: Allergy Specific (WPAI:AS) [50]. In addition, MASK-air users can set up their profile and provide information such as the type of AIT they are under (e.g., subcutaneous AIT, sublingual tablets AIT, sublingual drops AIT, etc.) as well as the allergens targeted by the AIT. 

MASK-air has enabled the collection of large amounts of direct patient data (formerly called “real-world data”), whose analysis has prompted several scientifically relevant achievements, such as (i) the joint impact of climate change, air pollution, and pollen season on allergic rhinitis [27], (ii) the finding that adherence to allergy treatment is poor [51] and do not follow guidelines but rather use rhinitis medication according to their symptoms [52], (iii) the identification of potential rhinitis phenotypes [29], and (iv) the development of a combined symptom-medication score (CSMS) measuring the daily control of allergic rhinitis [33]. Specifically regarding AIT, the analysis of MASK-air direct patient data has been the target of two cross-sectional studies – a proof-of-concept analysis and a study based on a Bayesian mixed-effects model. 

The first study involved the analysis of 317,176 days of MASK-air use (from 17,870 different users), of which 11% involved the reporting of AIT [41]. Days of users treated with AIT were found to display a lower median global allergy symptoms VAS than those of users not treated with AIT (9 vs. 12). Such differences were also observed (i) when separately considering days under no medication, days with single medication and days with co-medication and (ii) when comparing the levels of work VAS (instead of the global allergy symptoms VAS). This study did, however, display relevant limitations beyond the classical ones of mHealth studies. In particular, comparisons between days with vs. without AIT were not clustered by patient, were not adjusted for potential confounders, and did not take different types of AIT into account. 

Therefore, a subsequent study was conducted, assessing only grass allergy patients from ten European countries [42]. A total of 42,756 days from 1,093 patients were analyzed. The VASs and the CSMS from days of patients with vs. without AIT were compared, and the following factors were taken into account: (i) the clustering of observations by users, by countries, and by seasons and (ii) the adjustment for patients’ sex, age, and comorbidities (presence of asthma and conjunctivitis). We observed that patients treated with sublingual AIT (tablets) displayed a lower global allergy symptoms VAS, work VAS, and CSMS when compared to patients treated with subcutaneous AIT or not receiving AIT. By contrast, no differences were observed between patients treated with subcutaneous AIT vs. those not receiving AIT. Observed results were robust to analyses stratified by country or by the pollen season. 

Despite their different approaches, these studies obtained consistent results, suggesting that patients under AIT (particularly sublingual tablet AIT) report less severe allergy symptoms than those under no AIT. Consistent findings were observed when assessing the impact of allergic rhinitis in academic productivity, with the use of AIT being associated with a lower impact of rhinitis symptoms [43]. While several hypotheses can be postulated to explain this finding – from the effectiveness of AIT to the importance of differences in AIT types –, it should be noted that cross-sectional mHealth studies can only be used to generate new hypotheses. Such hypotheses should then be tested in future experimental and classical observational prospective studies. 

### AllergyMonitor as an example 

AllergyMonitor is an online service that was developed in 2009 with the aim of (i) enabling the recording of clinical symptoms, drug use, and adherence to AIT, and (ii) monitoring the efficacy of sublingual or subcutaneous AIT by patients with allergic rhino-conjunctivitis and/or asthma. The system, available to everyone and simple to use, consists of two parts: a patient app (front end) and a website for the attending doctor (back-office) [11]. The mobile app and back-office of AllergyMonitor allow patients to record their daily allergy symptoms, their drug and AIT intake, and any possible side effects in a customizable way. The results can be accessed by the patient and attending physician via a smartphone or computer. A concise report can be obtained in a collaborative setting of blended care. Geolocation is optional. This technology has been used since 2009 in several clinical studies and in routine practice. 

The download and usage of this app are free of charge. It falls under Italian jurisdiction, is CE1 registered, and follows the GDPR. The Technology Readiness Level (TRL) has been assessed for this app. It is available in 14 countries (TRL9) and contains the CARAT questionnaire as well as information on pollen counts (TRL9). The quality of the AllergyMonitor data was checked by estimating the percentage of changes in trends of the trajectories produced by the patients’ data [53]. 

The AllergyMonitor studies showed that (i) the etiological diagnosis of seasonal allergic rhinitis may be supported by prospectively matching registered symptoms with pollen counts, (ii) it is possible to perform a short-term prediction of rhinitis symptoms at individual level, (iii) the adherence to daily symptom monitoring can remain high (> 80%) throughout several weeks when prescribed and thoroughly explained by the treating doctor, (iv) the use of mobile technology can improve adherence to symptomatic drugs, and (v) the choice of the correct symptom-severity score is critical at patient level, but not at group level [11]. Particularly regarding AIT, an AllergyMonitor study assessing a cohort of 28 patients has found that the use of mobile technology can improve adherence to AIT. 

Most patients with pollen-induced allergic rhinitis are polysensitized. Therefore, an adequate definition of pollen seasons is essential for an optimal identification and management in allergic rhinitis patients [54, 55]. The @IT-2020 study is targeted to support etiologic diagnostics and AIT prescriptions for patients with seasonal allergic rhinitis. In the @IT.2020 multi-center study, pollen counts were collected over the course of 1 year (2018) in 6 Mediterranean cities [56]. The AllergyMonitor app improved the precision in diagnosing pollen allergy using daily symptom monitoring and graphical representations of airborne pollen data [56, 57].

## Symptom-medication scores using mHealth 

Symptom-medication scores are needed to investigate the efficacy of AIT, particularly in the clinical practice or as endpoints for RCTs [58, 59] and observational studies. The European Academy of Allergy and Clinical Immunology (EAACI) proposed the development of CSMSs for AIT trials [60]. The development of such CSMSs was based on tools that did not include symptoms or medications and that were associated with the social and/or economic impact of rhinitis. Such tools include, among others, work productivity and quality of life. 

MASK-air data were used for the development and assessment of such CSMSs. In particular, it allowed for the assessment of the concurrent validity, test-retest reliability, and responsiveness of one hypothesis-driven CSMS (modified CSMS: mCSMS), one mixed hypothesis- and data-driven score (mixed score), and several data-driven CSMSs generated by cluster analysis and regression models or factor analysis. These CSMSs were compared with scales measuring (i) the impact of rhinitis on work productivity (work VAS of MASK-air and WPAI-AS), (ii) quality of life (EQ-5D VAS), and (iii) control of allergic diseases (CARAT) [34, 41]. 

CSMSs can be used to stratify patients for AIT and to follow the patient during AIT (and beyond, when the treatment has ended) in RCTs and real-life observational studies. In addition, they can be combined with the pollen and air pollution data of the geolocalized patient, making it possible to correlate AIT effectiveness with daily allergen and pollution exposure [61].

## Closing remarks 

Mobile health tools allow the collection of large volumes of real-world data related to AIT. They have the potential to advance knowledge and improve the clinical practice in a patient-centered way (Box 1). In particular, we identified two mHealth apps which have assessed patients with AIT – MASK-air and AllergyMonitor – and which have enabled the evaluation of patients’ reported symptoms, the study of adherence, and the development of CSMSs. Nevertheless, mHealth studies have important limitations, namely related to the representativeness of app users, to the quality of self-reported information, and to the cross-sectional nature of analyses (Table 3). Therefore, these studies do not replace but rather complement more traditional epidemiological studies and RCTs. Future studies may use mHealth data to (i) assess how the use of AIT influences the impact of allergic rhinitis in work productivity and quality of life, (ii) contribute to health economic evaluation studies, (iii) better define pollen seasons to support an improved prescription of AIT, and (iv) raise hypotheses on possible shorter-term effects of AIT (Box 2). 

## Funding 

No specific funding was obtained for this article. 

## Conflict of interest 

JB reports personal fees from Chiesi, Cipla, Hikma, Menarini, Mundipharma, Mylan, Novartis, Sanofi-Aventis, Takeda, Teva, Uriach, other from KYomed-Innov; and personal fees from Purina, other from MASK-air. 

OP reports grants and personal fees from ALK-Abelló, Allergopharma, Stallergenes Greer HAL Allergy Holding B.V./HAL Allergie GmbH, Bencard Allergie GmbH/Allergy Therapeutics, Lofarma, ASIT Biotech Tools S.A., Laboratorios LETI/LETI Pharma, Anergis S.A., GlaxoSmithKline; personal fees from MEDA Pharma/MYLAN, Mobile Chamber Experts (a GA2LEN Partner), Indoor Biotechnologies, Astellas Pharma Global, EUFOREA, ROXALL Medizin, Novartis, Sanofi-Aventis and Sanofi-Genzyme, Med Update Europe GmbH, streamedup! GmbH, John Wiley and Sons, AS, Paul-Martini-Stiftung (PMS), Regeneron Pharmaceuticals Inc., RG Aerztefortbildung, Institut für Disease Management, Springer GmbH, AstraZeneca, IQVIA Commercial, Ingress Health; grants from Pohl-Boskamp, Inmunotek S.L., Biomay, Circassia. 

BSP reports no conflict of interest. 

**Table 1.** Summary description of the mHealth apps for allergic rhinitis with published results in the scientific literature [10, 40].

	AllergyMonitor	AirRater	MASK-air	Husteblume	Pollen
Countries	14	1	28	1	7
Published compliance with the GDPR	YES	NO	YES	NO	NO
List of all medications	YES, by medication and dosage, customized by country	YES	YES, by medication, customized by country	YES	YES, by drug class
Publications on allergen immunotherapy	[11]	NO	[41, 42, 43]	NO	NO

GDPR = General Data Protection Regulation.

**Table 2.** Visual analogue scales available in MASK-air.

VAS	Question
VAS global allergy symptoms	Overall, how much are your allergic symptoms bothering you today?
VAS nose	How much are your nose symptoms bothering you today?
VAS eyes	How much are your eye symptoms bothering you today?
VAS asthma	How much are your asthma symptoms bothering you today?
VAS work	How much are your allergic symptoms affecting your work today?
VAS school	Today, how much did allergies affect your productivity while in school or attending classes in an academic setting?

VAS = visual analogue scale.

**Box 1.** Take-home messages on mHealth apps in the context of allergen immunotherapy for allergic rhinitis.

– Despite the potential of mHealth in monitoring patients with AIT, there are only two apps with scientific publications that can be used in patients with allergic rhinitis and undergoing AIT: MASK-air and AllergyMonitor. – In clinical practice, the use of mHealth apps allows for a better monitoring of patients with allergic rhinitis taking AIT (through the data patients regularly provide directly in the apps) and for increased adherence to AIT. – From a scientific point of view, mHealth apps can be used to generate hypotheses that can then be complemented by classical epidemiological studies and/or randomized controlled trials. Data from mHealth apps have suggested that (i) a better control of rhinitis symptoms in patients under AIT appears to occur irrespective of the medication taken by the patient, and (ii) sublingual AIT is associated with better rhinitis control when compared to subcutaneous AIT.

AIT = allergen immunotherapy.

**Table 3.** Main benefits and limitations of using mHealth apps in the context of allergen immunotherapy.

Benefits	Limitations
– Possibility of collecting regular data from patients, allowing them to be monitored on a daily basis regarding their allergic rhinitis symptoms and impact. – Adoption of patient-centered strategies, allowing patients to better understand and self-manage their disease. – Collection of large volumes of data from “real-world” patients at a low cost, overcoming some limitations of classical randomized controlled trials (highly-selected participants and high costs). – Advancing scientific knowledge on AIT, namely through (i) the development of scores assessing disease control, (ii) the assessment of patient-reported symptoms in relation to AIT, and (iii) the assessment of rhinitis impact on patients’ lives and its relationship with AIT use.	– mHealth apps may not be adequate for all patients, possibly requiring more effort for older patients or for those with lower digital literacy. Therefore, patients using mHealth apps may not be representative of the general population. – Data daily provided by patients in mHealth are not subject to physician confirmation, leading to possible limitations in the quality of self-reported information. – Adherence to app use is not high for many patients, impairing their longitudinal assessment (e.g., to assess the effectiveness of AIT in each individual patient). – Impossibility of retrieving data on all relevant confounders, limiting the capacity to assess the effectiveness of AIT

AIT = allergen immunotherapy.

**Box 2.** Future studies that may be conducted with mHealth data regarding allergen immunotherapy for allergic rhinitis.

– Assessment on how the use of immunotherapy influences the impact of allergic rhinitis in work productivity, sleep quality, and quality of life. – Health economic evaluation studies using direct patient data provided through mHealth apps, both to estimate resource use (e.g., use of AIT and medication) and the impact of AIT on work productivity and quality of life (e.g., with computation of utilities). – Better definition of pollen seasons (combining environmental and direct patient data) to support an improved prescription of AIT. – Assessment of possible short-term effects of AIT.

AIT = allergen immunotherapy.
